# Lack of pathogenic potential of peripheral α-synuclein aggregates from Parkinson’s disease patients

**DOI:** 10.1186/s40478-018-0509-1

**Published:** 2018-02-08

**Authors:** Ariadna Recasens, Iria Carballo-Carbajal, Annabelle Parent, Jordi Bové, Ellen Gelpi, Eduardo Tolosa, Miquel Vila

**Affiliations:** 1 Neurodegenerative Diseases Research Group, Vall d’Hebron Research Institute-Center for Networked Biomedical Research on Neurodegenerative Diseases (CIBERNED), 08035 Barcelona, Spain; 2 Neurological Tissue Bank, Biobanc Hospital Clínic-IDIBAPS, 08036 Barcelona, Spain; 3 Institute of Neurology, Medical University of Vienna, 1090 Vienna, Austria; 4 Parkinson’s Disease and Movement Disorders Unit, Institut Clínic de Neurociències, Hospital Clinic of Barcelona-CIBERNED, 08036 Barcelona, Spain; 5 Department of Biochemistry and Molecular Biology, Autonomous University of Barcelona, 08193 Barcelona, Spain; 6 Catalan Institution for Research and Advanced Studies (ICREA), 08010 Barcelona, Spain

## Abstract

In Parkinson’s disease (PD) there is widespread accumulation in the brain of abnormal α-synuclein aggregates forming intraneuronal Lewy bodies (LB). It is now well established that LB-type α-synuclein aggregates also occur in the peripheral autonomic nervous system in PD, from where it has been speculated they may progressively spread to the central nervous system through synaptically-connected brain networks and reach the substantia nigra to trigger herein dopaminergic dysfunction/degeneration and subsequent parkinsonism. Supporting a pathogenic role for α-synuclein aggregates we have previously shown that LB purified from postmortem PD brains promote α-synuclein pathology and dopaminergic neurodegeneration when intracerebrally inoculated into wild-type mice. However, the pathogenic capacity of PD-derived peripheral α-synuclein aggregates remains unknown. Here we addressed this question using purified LB-type α-synuclein aggregates from postmortem PD stellate ganglia (SG), a paravertebral sympathetic ganglion that exhibits consistent and conspicuous Lewy pathology in all PD patients. In contrast to our previous findings using nigral LB extracts, intracerebral inoculation of SG-derived LB into mice did not trigger long-term nigrostriatal neurodegeneration nor α-synuclein pathology. The differential pathogenic capacities of central- and peripheral-derived α-synuclein aggregates appear independent of the absolute amount and basic biochemical properties of α-synuclein within these aggregates and may rely instead on differences in α-synuclein conformation and/or yet unrecognized brain region-specific intrinsic factors. Our results argue against a putative pathogenic capacity of peripheral α-synuclein aggregates to promote α-synuclein pathology in the brain, propagate between neuronal networks or induce neurodegeneration.

**Electronic supplementary material:**

The online version of this article (10.1186/s40478-018-0509-1) contains supplementary material, which is available to authorized users.

## Introduction

Brain pathology in Parkinson’s disease (PD) is characterized by prominent loss of dopaminergic neurons from the substantia nigra pars compacta (SNpc) and the presence in affected brain regions of intraneuronal Lewy bodies (LB) and Lewy neurites (LN) consisting mostly of aggregated α-synuclein. At late stages of the disease, deposits of aggregated α-synuclein are widely spread in the central nervous system (CNS). In addition, it is now well established that LB-type α-synuclein aggregates also occur in the peripheral autonomic nervous system (pANS) [1, 8, 25]. It has been proposed that Lewy pathology may actually start in the periphery and progressively spread to the CNS through synaptically-connected brain networks, driving neuronal dysfunction and death along the way [9]. Supporting this concept, most PD patients experience a premotor period several years before the emergence of parkinsonism characterized by a variety of non-motor symptoms driven by neuronal damage in extranigral regions of the central and peripheral autonomic nervous system [22]. Furthermore, α-synuclein brain pathology can be triggered in experimental animals, including rodents and non-human primates, by intracerebral, intramuscular, intragastrical or intravenous inoculation of recombinant α-synuclein fibrils or brain homogenates from affected mice and patients [5, 11, 13, 14, 16, 18–21]. Indeed, we have previously shown that intranigral inoculation of α-synuclein-containing LB extracts purified from the SNpc of PD brains promotes widespread α-synuclein pathology and dopaminergic neurodegeneration in recipient mice [20]. However, the pathogenic capacity of PD-derived peripheral α-synuclein aggregates remains unknown.

Here we assessed for the first time the in vivo pathogenic potential of peripheral α-synuclein aggregates by inoculating α-synuclein-containing LB extracts purified from postmortem stellate ganglia (SG) of PD patients into the SNpc of wild-type mice. SG is a paravertebral sympathetic ganglion that exhibits consistent and conspicuous Lewy pathology in PD patients, but not in control subjects [7, 8, 17]. In fact, SG is one of the few peripheral tissues that invariably exhibits α-synuclein pathology in PD and the one with the highest burden of pathological α-synuclein [8]. In particular, abundant LB and LN-like structures immunoreactive for α-synuclein, phosphorylated α-synuclein and ubiquitin are seen in the SG of PD patients, with a highest density at the periphery of the ganglia [8]. Lewy pathology in the SG is accompanied by neurodegenerative changes in this ganglion, such as enlarged neurons with reduced tyrosine hydroxylase (TH) immunoreactivity, loss of pigmented and non-pigmented neurons with increased cellularity and formation of Nageotte nodules, as well as frequent microglia/macrophage activation [8]. For the above reasons, here we have chosen the SG to purify PD-linked peripheral α-synuclein aggregates to assess their pathogenic potential in vivo.

## Methods

### Purification of LB from human SG

Human fresh frozen postmortem SG samples from three patients with sporadic PD and two non-PD control subjects were obtained from the Neurological Tissue Bank, *Biobanc Hospital Clínic*-IDIBAPS (see Table 1 for details). SG is a sympathetic ganglion located in front of the neck of the first rib (at the level of the 7th cervical vertebrae) formed by the fusion of the inferior cervical and 1st thoracic (T1) sympathetic ganglia. SG samples were homogenized in 9 vol (*w*/*v*) ice-cold MSE buffer (10 mM MOPS/KOH, pH 7.4, 1 M sucrose, 1 mM EGTA, and 1 mM EDTA) with protease inhibitor cocktail (Complete Mini; Boehringer Mannheim) with 12 strokes of a motor-driven glass/teflon homogenizer. For LB purification, a sucrose step gradient was prepared by overlaying 2.2 M with 1.4 M and finally with 1.2 M sucrose in volume ratios of 3.5:8:8 (*v*/v), as previously described [20]. The homogenate was layered on the gradient and centrifuged at 160,000 x *g* for 3 h using a SW32.1 rotor (Beckman). Twenty fractions of 500 μl were collected from each gradient from top (fraction 1) to bottom (fraction 20), and analyzed for the presence of α-synuclein aggregates by filter retardation assay.

**Table 1:** Human sample information

Case	Gender	Age at death (years)	Diagnosis	aSyn in SG	PMI
PD#1	Female	84	PD stage 5	positive	4.5
PD#2	Female	81	PD stage 5	positive	6.5
PD#3	Male	68	PD stage 4	positive	9.33
Ctrl#1	Female	89	AD VI C+ TDP amygdala	negative	4.25
Ctrl#2	Male	88	AD IV C + thalamic infarct	negative	18.25

*PD* Parkinson’s disease, *AD* Alzheimer type pathology according to Braak staging (I-VI) and CERAD criteria (A-C), *TDP* Transactive response DNA binding protein 43 kDa, *aSyn* α-synuclein, *SG* stellate ganglion, *PMI postmortem* interval (hours)

### Filter retardation assay

This technique was performed as previously described [20]. Briefly, after heating at 100 °C for 5 min, samples (70 mg) were diluted in 200 μl of migration buffer (25 mM Tris-HClBase, 200 mM glycine, SDS 1%) and filtered through a cellulose acetate membrane (Schleicher & Schuell; 0.2 μm pore size) using a *Minifold-1 Dot-Blot System* (Schleicher & Schuell). Membranes were saturated in 5% dried skimmed milk in phosphate-buffereld saline (PBS) and probed with human α-synuclein antibody (see below for details). Appropriate secondary antibodies coupled to peroxidase were revealed using a Super Signal West Pico Chemiluminescent kit (Pierce). Chemiluminescence images were acquired using the ImageQuant RT ECL Imager (GE Healthcare).

### Immunoblot

SG-derived fractions were resolved by SDS-PAGE on 15% polyacrylamide gels and electrotransferred onto nitrocellulose membranes (GE Healthcare, #10401196), then blocked in 5% non-fat milk powder in tris-buffered saline (TBS) containing 0.1% tween-20 (TBS-T) for 1 h at room temperature (RT) and incubated overnight at 4 °C with a mouse anti-human α-synuclein antibody (1/500 in 4% bovine serum albumin/TBS-T, Thermo Scientific, #MS1572). Incubation with the anti-mouse secondary antibody coupled to horseradish peroxidase (1:2000 in 5% non-fat milk powder/TBS-T; Amersham Biosciences, #NXA931) was performed at RT for 1 h, followed by repeated washings with TBS-T. Immunoreactive bands were visualized using SuperSignal Femto Chemiluminescent Substrate (Pierce, #34096) according to the manufacturer’s instructions on an ImageQuant RT ECL imaging system (GE Healthcare).

### Enzyme-linked immunosorbent assay

Total protein concentrations in the different sucrose extracts were determined using the bicinchoninic acid assay (#23227; Thermo Scientific, Waltham, MA). Samples were bath sonicated for 5 min, diluted to 20 μg/ml of total protein and analyzed in triplicates for total α-synuclein protein levels with a specific enzyme-linked immunosorbent assay (ELISA) kit against human α-synuclein (Life Technologies, #KHB0061) according to the manufacturer’s instructions.

### Stereotactic inoculations

**Fig. 1:**
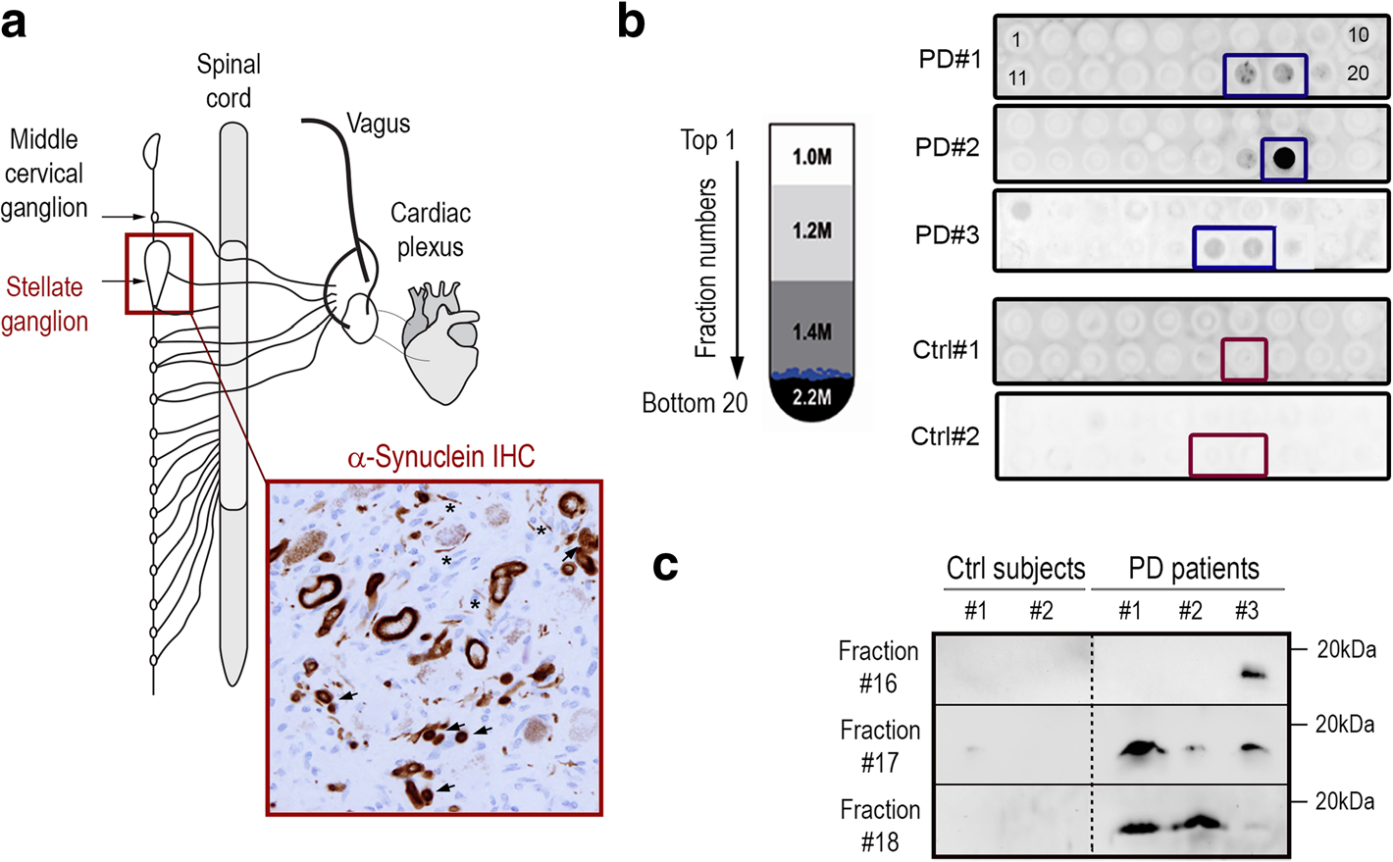
Purification of peripheral Lewy bodies (LB) from stellate ganglion (SG) of PD patients. **a** Schematic representation of SG localization and representative photomicrograph of α-synuclein pathology by immunohistochemistry (in brown) in the SG of one of the PD patients used in this study, as previously reported [8]. *Arrows*, LB-like structures; *asterisks*, Lewy neurites. **b** *Left*, schematic representation of the sucrose gradient fractionation procedure used to purify LB-containing fractions from freshly frozen human SG tissue. *Right*, filter retardation assay probed with a human α-synuclein antibody to assess the presence of insoluble α-synuclein aggregates in the different fractions obtained by sucrose gradient fractionation from the SG of three sporadic PD patients and two non-PD control (Ctrl) subjects. Rectangles indicate the fractions selected to prepare the mixture used for inoculations. **c** Immunoblot levels of human α-synuclein in fractions 16 to 18 (corresponding to the 1.4/2.2 M interface) from PD patients and non-PD control subjects

For stereotactic inoculations, LB-containing SG fractions from the three PD patients were first mixed together in the same proportion (PD #1, fractions 17 and 18; PD #2, fraction 18; PD #3, fractions 16 and 17; see Fig. 1). Control animals were inoculated with a mixture of equivalent fractions obtained from SG of two age-matched human control subjects lacking α-synuclein pathology (Ctrl #1, fraction 17; Ctrl #2, fractions 16 and 17; see Fig. 1). An additional control group was injected with the corresponding buffer (vehicle) obtained from a sucrose gradient purification performed without the addition of any human tissue sample. In all cases, samples were bath-sonicated for 5 min prior to the in vivo inoculations. Wild-type C57BL/6 mice (3 months old) received 2 μl of either LB-SG fractions, control-derived fractions, or appropriate buffer (vehicle) by stereotactic delivery to the region immediately above the right substantia nigra (− 2.9 mm AP, 1.3 mm L and − 4.5 mm DV) at a flow rate of 0.4 μl/min. Animals were euthanized at either 24 h or 6 months after injection and the position of the 30-gauge needle was determined histologically.

### Cylinder behavioral test

Mice were tested for left and right forepaw use with the cylinder test one week before surgery (to establish the basal conditions for each animal) and at 6 months post-surgery. For the performance of the cylinder test, mice were first allowed to habituate to the experimental room for at least one hour before each test. Then, mice were put in a glass cylinder and the total number of left and right forepaw touches performed within 5 min was counted. Data are presented as the percentage of contralateral forepaw use with respect to total forepaw use (contralateral + ipsilateral). All behavioral tests were performed during the light cycle by an investigator blinded to the experimental groups.

### Immunohistochemistry

Twenty-four hours or six months after stereotactic inoculations, mice were euthanized by perfusion with 4% PFA and their brains were processed for immunohistochemical analyses. Immunostaining was performed on 20 μm-thick free-floating sections incubated with different primary antibodies (see below) for 24 h at 4 °C. Biotinylated secondary antibodies, followed by signal amplification using the avidin-biotin complex (ABC) method, were used. Immunostaining was revealed using 3,3′-diaminobenzidine tetrahydrochloride (DAB, Sigma Aldrich, #D5905-50TAB). When mouse secondary antibodies were required, a Vector M.O.M Immunodetection kit (Vector Laboratories, #BMK2202) was used, according to the manufacturer’s instructions. The following antibodies were used: Tyrosine Hydroxylase (1/1000, Calbiochem, #657012), human α-synuclein Ab-2 (clone syn211, 1/250, Thermo Scientific, #MS-1572), α-synuclein (1/1000, BD Transduction Laboratories, #610786), phosphorylated α-synuclein (phospho S129, 1/750, Abcam, #2014–1), Ionized calcium binding adaptor molecule 1 (Iba1, 1/1000, Wako Pure Chemical Industries, #019–19,741) and Glial Fibrillary Acid Protein (GFAP, 1/1000, Sigma-Aldrich, #G3893).

### Quantitative morphology

*Nigrostriatal integrity*: the total number of TH-positive SNpc neurons was assessed by stereology with the optical fractionator method in regularly spaced (every sixth) 20 μm-thick sections spanning the entire SNpc using StereoInvestigator software (MBF Bioscience). Striatal TH innervation was assessed by optical densitometry in regularly spaced 20 μm-thick sections corresponding to different striatal anatomical levels using Sigma Scan.

*α-Synuclein pathology*: (1) the total number of α-synuclein-positive neurons in the SNpc was assessed by stereology, as above; (2) the total number of hyperphosphorylated α-synuclein (pSyn)-positive neurons in the SNpc was manually assessed under light microscopy in four sections spanning the entire SN; (3) pSyn at regional level was assessed by optical densitometry using the ImageJ software in pSyn-immunostained sections scanned with an Epson Perfection V750 PRO scanner (Long Beach, CA); (4) intracellular α-synuclein levels were assessed by optical densitometry at cellular level using Sigma Scan in transmitted-light microscopy images of α-synuclein-positive cells from the SNpc (~ 650 neurons per group, randomly selected among the different animals).

*Neuroinflammation*: for inflammatory reaction assessment, microglia and astrocyte density were estimated by OD at regional level in Iba1- and GFAP-immunostained sections, respectively, using Sigma Scan software. All analyses were performed blinded to the researcher.

### Proteinase K digestion

Twenty-micrometer-thick sections were washed with TBS and incubated in proteinase K (PK; Invitrogen, Carlsbad, CA; 1 μg/ml in TBS) at RT for 10 min. The sections were then washed in TBS and immunostained for α-synuclein as indicated above.

### Statistical analysis

All values are expressed as the mean ± standard error of the mean (SEM). Statistical comparisons between control-injected and LB-SG-injected animals were performed with SigmaStat software (v4, Systat Software Inc., USA) using two-tailed t-test, Mann-Whitney rank sum test or one-way ANOVA, as appropriate. Selection of the pertinent statistical test for each experiment was determined after formally testing for normality. In all analyses, the null hypothesis was rejected at the 0.05 level.

## Results

### Purification of SG LB from PD patients

Peripheral LB-containing fractions were obtained from freshly frozen postmortem SG tissue derived from 3 patients with sporadic PD exhibiting abundant LB pathology at neuropathological examination (Fig. 1a). In the SG of these patients, Lewy pathology appeared in the form of α-synuclein immunopositive LB-like structures and LN (Fig. 1a), as previously described [8]. Two main types of LB-like structures could be observed in these samples: (i) filamentous LB with a dense central core and radiating filaments, similar to the classical LB found in the SN, with a stronger ring-like α-synuclein immunoreactivity at the periphery than in the cores (Fig. 1a) and (ii) ill-defined structures that were diffusely immunoreactive for α-synuclein. These structures were previously found to contain also hyperphosphorylated pathological forms of α-synuclein (phosphorylated at serine 129) and ubiquitin, two major components of LB [8]. In contrast, no α-synuclein pathology was observed in the SG of age-matched non-PD control subjects (Additional file 1: Figure S1). LB purification from PD-derived SG samples was achieved by sucrose gradient fractionation, as previously done for SNpc-derived LB purification [20]. Briefly, SG samples were homogenized and layered on the top of a sucrose gradient (from 1.0 M to 2.2 M), with LB-containing fractions being recovered at the 1.4/2.2 M interface after ultracentrifugation (Fig. 1b). To identify LB-containing fractions, all recovered sucrose gradient fractions (*N* = 20) were screened by filter retardation assay for the presence of insoluble α-synuclein aggregates (Fig. 1b), as previously validated [20]. Using this biochemical assay, LB fractions containing insoluble α-synuclein aggregates were found within fractions 16 to 18, as expected (Fig. 1b). None of the fractions derived from SG samples of non-PD control subjects contained α-synuclein aggregates (Fig. 1b). The presence of α-synuclein in PD-derived, but not control-derived, SG fractions was further confirmed by immunoblot (Fig. 1c). For stereotactic inoculations to mice, LB-containing SG fractions from the 3 PD patients were mixed together in the same proportion, as follows: PD #1, fractions 17 and 18; PD #2, fraction 18; PD #3, fractions 16 and 17 (Fig. 1b). Prior to inoculation to mice, this mixture (called “LB-SG” henceforth) was bath-sonicated for 5 min, to disrupt the aggregates into fibrillar fragments of different sizes, as in our previous study using SNpc-derived LB fractions [20]. Quantifications by ELISA indicated that this mix contained 17.27 ± 2.23 ng of α-synuclein per milligram of total protein, similar to the ~ 15 ng/mg previously obtained from SNpc-derived LB fractions [20]. Control animals were injected with equivalent fractions obtained from SG of age-matched non-PD human control subjects lacking α-synuclein pathology (Fig. 1b, c). An additional control group was injected with the corresponding buffer (vehicle) obtained from a sucrose gradient purification performed without the addition of any human tissue sample.

### Lack of nigrostriatal degeneration in mice inoculated with SG-derived LB extracts

**Fig. 2:**
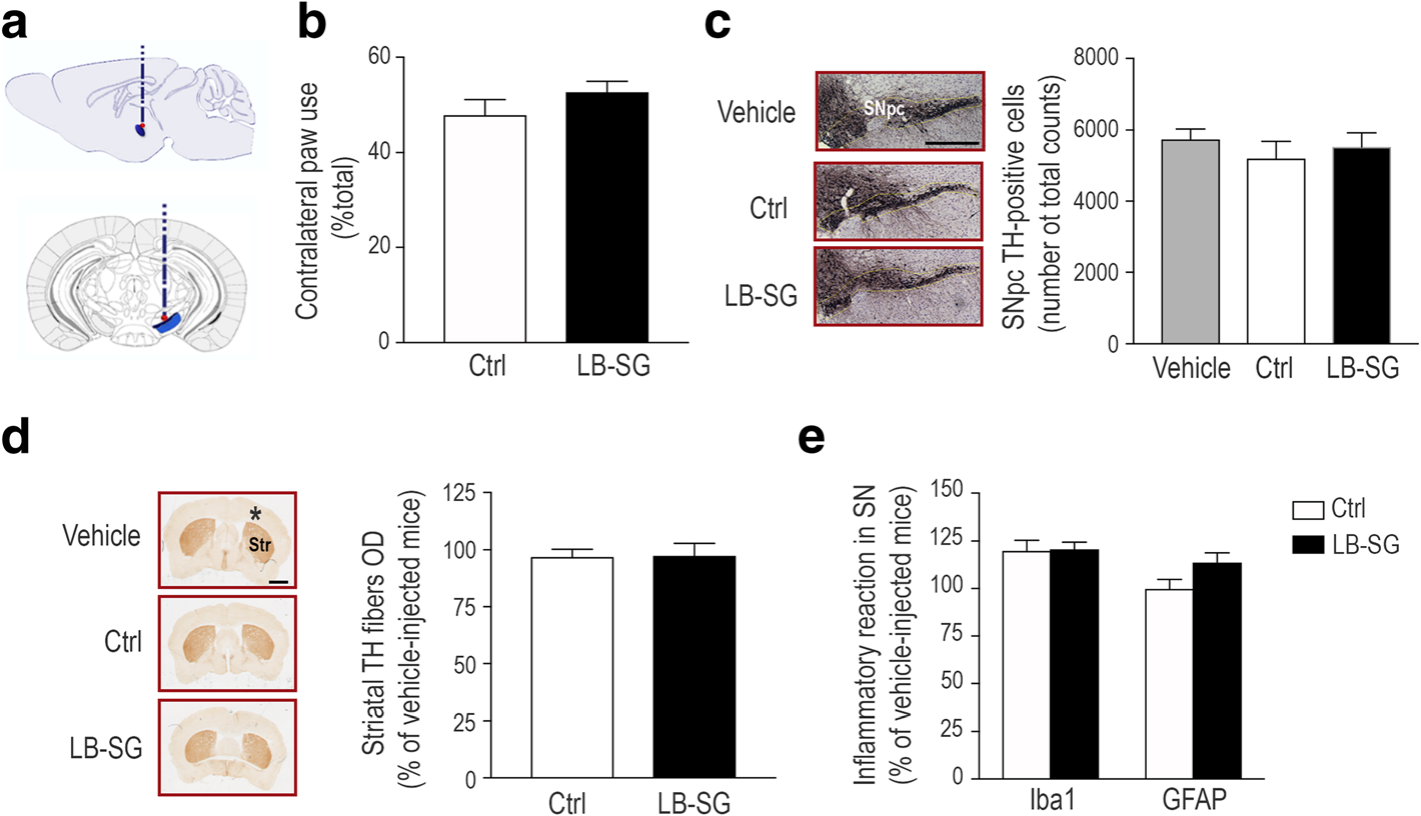
Integrity of the nigrostriatal pathway in LB-SG-injected mice. **a** Schematic diagram indicating the site of nigral stereotactic inoculations to mice. **b** Contralateral forepaw use, as assessed by the cylinder test, in mice injected with control- and PD-derived LB-SG fractions at 6 months post-inoculation. *Ctrl*: *n* = 8; mean = 47.87; SD = 9.128; SEM = 3.227; *LB-SG*: *n* = 12; mea*n* = 52.62; SD = 7.929; SEM = 2.289; *p* = 0.23, two-tailed t-test. **c** *Left*, representative photomicrographs of ipsilateral tyrosine hydroxylase (TH)-positive substantia nigra pars compacta (SNpc, outlined in red) neurons (dark brown; thionin staining in purple) from vehicle-, Ctrl- and LB-SG-injected animals, at 6 months post-inoculation. *Right*, quantification of ipsilateral TH-positive SNpc neurons by stereology in vehicle-, Ctrl- and LB-SG-injected mice, at 6 months post-inoculations. *Vehicle*: *n* = 9; mean = 5736.66; SD = 879.61; SEM = 293.20; *Ctrl*: *n* = 7; mean = 5198.57; SD = 1282.38; SEM = 484.69; *LB-SG*: n = 9; mean = 5520; SD = 1219.34; SEM = 406; *p* = 0.644, one-way ANOVA. **d** *Left*, representative photomicrographs of TH-positive striatal terminals (in brown) from vehicle-, Ctrl- and LB-SG-injected animals at 6 months post-inoculation (asterisk indicate injected hemisphere). *Right*, quantification of ipsilateral TH-positive striatal terminals by optical densitometry (OD) in Ctrl- and LB-SG-injected mice, relative to vehicle-injected animals, at 6 months post-inoculations. *Ctrl*: n = 8; mean = 96.86; SD = 9.52; SEM = 3.37; *LB-SG*: *n* = 11; mean = 97.39; SD = 18; SEM = 5.427; *p* = 0.836, Mann-Whitney rank sum test. **e** Quantification of microglial Iba1 and astrocytic GFAP reactions by OD in the ipsilateral SN of Ctrl- and LB-SG-injected mice, relative to vehicle-injected animals, at 6 months post-inoculations. For Iba1, *Ctrl*: n = 8; mean = 119.8; SD = 16.04, SEM = 5.671*; LB-SG*: n = 11; mean = 120.7; SD = 12.56; SEM = 3.788; *p* = 0.773 Mann-Whitney rank sum test. For GFAP, *Ctrl*: n = 8; mean = 99.77; SD = 14.3; SEM = 5.055; *LB-SG*: n = 12, mean = 113.7; SD = 17.67; SEM = 5.101; *p* = 0.08, two-tailed t-test. In all panels, histograms represent average ± standard error of the mean (SEM). Vehicle-injected mice, n = 9–11. Scale bars, 500 μm (**c**) and 1 mm (**d**)

To determine the potential pathogenic effects of peripheral α-synuclein aggregates from PD patients, adult wild-type C57BL6 mice received single unilateral stereotaxic inoculations (2 μl) of either LB-SG fractions, control-derived fractions, or vehicle immediately above the right SNpc (Fig. 2a), as we have previously done for SNpc-derived LB fractions [20]. Six months after inoculation, all animal groups were subjected to behavioral and histological analyses. This time-frame was chosen based on previous studies indicating that a pathogenic effect of either synthetic recombinant α-synuclein pre-formed fibrils or SNpc-derived LB extracts from PD brains can already be observed by 3–4 months post-inoculation to mice [13, 20]. First, we assessed motor ability in LB-SG-injected mice using the cylinder test, which allows asymmetrical alterations in nigrostriatal dopaminergic function to be detected in unilaterally-injected mice [15]. Using this test, we did not observe any contralateral forepaw hypokinesia in LB-SG-injected mice, as indicated by an equivalent use of both forepaws in these animals, similar to control-injected groups (Fig. 2b). We next assessed the integrity of the dopaminergic nigrostriatal system in these animals, at the level of both SNpc cell bodies and striatal axon terminals, by stereological cell counts of dopaminergic TH-positive SNpc neurons and by optical densitometry of striatal dopaminergic TH-positive fibers, respectively. Consistent with the lack of motor impairment, no nigrostriatal degeneration was observed in LB-SG-injected animals at 6 months post-inoculation, as indicated by a preservation of both SNpc dopaminergic cell bodies and striatal dopaminergic fibers in these animals (Fig. 2c-d). In addition, no evidence of ongoing neuroinflammatory changes was observed in these animals, as shown by a lack of microglial and astrocytic reaction in their SNpc (Fig. 2e). Overall, our results indicate that, in contrast to what we have previously seen for SNpc-derived LB extracts [20], peripheral LB-SG extracts are not able to trigger nigrostriatal neurodegeneration when injected to mice.

### Lack of α-synuclein pathology in mice inoculated with SG-derived LB extracts

**Fig. 3:**
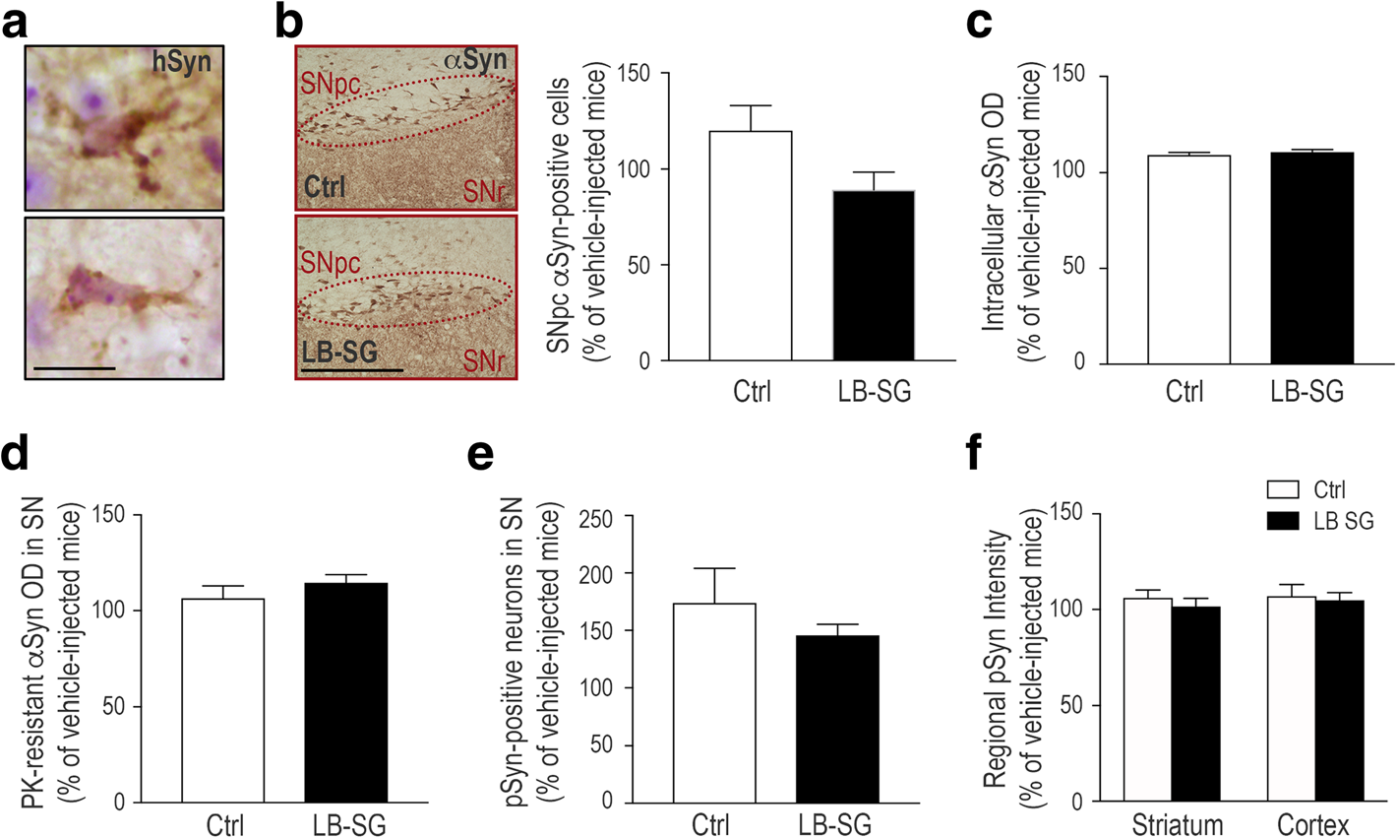
Lack of α-synuclein pathology in LB-SG-injected mice. **a** Immunohistochemistry of human α-synuclein (hSyn, in brown; thionin staining in purple) in the SNpc of LB-SG-injected mice at 24 h post-inoculation. **b** *Left*, representative photomicrographs of endogenous murine α-synuclein immunostaining (in brown) in the SN of Ctrl- and LB-SG-injected animals at 6 months post-inoculation. *Right*, number of α-synuclein-immunopositive neurons in the ipsilateral SNpc of Ctrl- and LB-SG-injected mice, relative to vehicle-injected animals, at 6 months post-inoculations. *Ctrl*: n = 5; mean = 119.8; SD = 29.38; SEM = 13.14; *LB-SG*: n = 9; mean = 89.14; SD = 27.94; SEM = 9.313; *p* = 0.0768, two-tailed t-test. **c** Quantification of intracellular levels of endogenous murine α-synuclein within ipsilateral SNpc neurons by optical densitometry (OD) in Ctrl- and LB-SG-injected mice, relative to vehicle-injected animals, at 6 months post-inoculations. *Ctrl*: *n* = 645; mea*n* = 109.3; SD = 37.06; SEM = 1.459; *LB-SG*: *n* = 603; mean = 110.8; SD = 34.27; SEM = 1.396; *p* = 0.32, Mann-Whitney rank sum test. **d** Quantification of regional levels of endogenous murine α-synuclein by OD in the ipsilateral SN of Ctrl- and LB-SG-injected mice at 6 months post-inoculations, relative to vehicle-injected animals, after proteinase K (PK) digestion (1 μg/ml for 10 min). *Ctrl*: n = 6; mean = 106.5; SD = 15.82; SEM = 6.458; *LB-SG*: n = 12; mean = 114.6; SD = 14.61; SEM = 4.218; *p* = 0.2959, two-tailed t-test. **e** Number of phosphorylated α-synuclein (pSyn)-immunopositive neurons in the ipsilateral SNpc of Ctrl- and LB-SG-injected mice, relative to vehicle-injected animals, at 6 months post-inoculations. *Ctrl*: n = 8; mean = 173.9; SD = 85.39; SEM = 30.19; *LS-SG*: n = 10; mean = 145.7; SD = 30.83; SEM = 9.749, *p* = 0.3435, two-tailed t-test. **f** Quantification of regional levels of pSyn by OD in the striatum and neocortical areas (i.e., cingulate, motor, and somatosensory cortices) of Ctrl- and LB-SG-injected mice, relative to vehicle-injected animals, at 6 months post-inoculations. For striatum, *Ctrl*: n = 7; mean = 106.1; SD = 10.69; SEM = 4.041; *LB-SG*: n = 10; mean = 101.5; SD = 13.6; SEM = 4.3; *p* = 0.47, two-tailed t-test. For cortex, *Ctrl*: n = 7; mean = 106.9; SD = 16.15; SEM = 6.105; *LB-SG*: n = 10; mean = 104.9; SD = 12.59; SEM = 3.981; *p* = 0.773, two-tailed t-test. In all panels, histograms represent average ± standard error of the mean (SEM). Vehicle-injected mice, n = 8–10. Scale bars, 30 μm (**a**) and 500 μm (**b**)

We next determined whether intranigral inoculation of SG-derived LB extracts could trigger α-synuclein pathology in injected animals, as we have previously seen for SNpc-derived LB extracts [20]. Using an antibody that recognizes human but not murine α-synuclein, we found that exogenously inoculated SG-linked human α-synuclein was internalized by host murine SNpc neurons by 24 h post-injection (Fig. 3a). At this time-point, exogenous human α-synuclein was detected in LB-SG-injected mice, but not in control-injected animals, as a punctate, inclusion-like immunolabeling within the cytoplasm and along the processes of murine host cells (Fig. 3a). No exogenous human α-synuclein immunosignal was detected in LB-SG-inoculated animals by 6 months post-inoculation.

Because we previously found that inoculation of SNpc-derived LB extracts from PD patients to mice triggered the pathological conversion and intracellular accumulation of endogenous murine α-synuclein [20], we next assessed whether injection of LB-SG fractions resulted in a similar pathogenic effect. Using an antibody that recognizes murine α-synuclein, we assessed first by stereology the number of α-synuclein-immunopositive neurons in the SNpc of LB-SG-injected mice at 6 months post-inoculation. No differences were found in the number of SNpc α-synuclein-positive neurons between LB-SG- and control-injected animals (Fig. 3b). Similarly, no differences were found in the intracellular levels of α-synuclein, assessed by optical densitometry, between LB-SG- and control-injected animals at 6 months (Fig. 3c). In the absence of changes in α-synuclein levels, we next assessed whether endogenous α-synuclein in LB-SG-injected mice might have adopted a pathological insoluble/aggregated beta-sheet conformation resistant to proteinase K (PK) digestion, as we have previously seen for SNpc-derived LB extracts [20]. Ruling out this possibility, no differences were found in the susceptibility of α-synuclein to PK digestion (1μg/ml for 10 min) between LB-SG- and control-injected animals at 6 months (Fig. 3d). Further excluding a pathological conversion of α-synuclein in LB-SG-injected mice, no changes in hyperphosphorylated pathological forms of α-synuclein were found between LB-SG- and control-injected animals at 6 months (Fig. 3e-f). LB-SG- and control-injected animals exhibited equivalent levels of pSyn, both at the site of injection (i.e. SNpc, Fig. 3e) and in distant anatomically connected brain regions (i.e. striatum and neocortical areas, Fig. 3f). Overall, these results indicate that, in contrast to nigral LB extracts [20], peripheral LB-SG extracts are not able to trigger long-term α-synuclein pathology in mice.

## Discussion

This study assessed the pathogenic potential of peripheral α-synuclein aggregates from PD patients. In contrast to our previous observation using nigral-derived LB extracts [20], peripheral α-synuclein aggregates from SG did not produce any pathologic effect when inoculated into the brain of recipient wild-type mice, up to 6 months post-injection. While exogenous human α-synuclein from SNpc- and SG-derived LB extracts were both quickly internalized by host murine SNpc neurons, only animals receiving SNpc-derived LB extracts exhibited PD-like pathology [20]. Indeed, in previous experiments, animals injected with SNpc-derived LB extracts exhibited nigrostriatal neurodegeneration and widespread α-synuclein pathology by 4 months post-inoculation [20], while none of which was observed in LB-SG-injected mice at 6 months. The differences in pathogenicity between SNpc- and SG-derived extracts cannot be attributed to methodological issues, as both studies were performed in an identical manner, including the intracerebral site of injection (i.e. SNpc) [20]. Both SNpc and SG LB fractions were purified using the same sucrose gradient fractionation method and the amount of total α-synuclein recovered in these fractions was comparable (~ 15 ng/mg vs ~ 17 ng/mg, respectively, as quantified by ELISA). In addition, the distribution pattern of α-synuclein aggregates, revealed by filter retardation assay, was equivalent between nigral and SG LB fractions, corresponding in both studies to recovered fractions #16–18. The differential pathogenicity did not result either from the presence of a putative pathogenic factor other than α-synuclein specifically within nigral-derived LB fractions, since the presence of α-synuclein in these fractions was an absolute requirement for their pathogenic effect [20]. The differential pathogenicity between nigral and SG LB fractions might be linked to differences in α-synuclein conformation within these fractions and/or to yet unrecognized region-specific intrinsic factors. Supporting this concept, crosslinking experiments have shown that endogenous α-synuclein species are different between human brain and human small intestine [4]. Along this line, a previous study in A53T α-synuclein-overexpressing transgenic mice reported that α-synuclein oligomers obtained from different CNS regions exhibited differential pathogenic capacities in vitro, in terms of promoting α-synuclein amyloid fibril formation and neurotoxicity, despite sharing similar biochemical properties [26]. It has also been recently revealed the existence of different strains of α-synuclein able to adopt different structural conformations that cause distinct histopathological and behavioral phenotypes when injected into experimental animals [19]. In this context, SG LB fractions might need further maturation (e.g. conformation changes, protein interactions, further processing and/or additional post-translational modifications beyond phosphorylation) to acquire pathogenic characteristics identical to those originating from SNpc. Alternatively, SG LB extracts might just be slower in triggering α-synuclein pathology and thus require longer incubation times than SNpc-derived α-synuclein aggregates to produce pathology. Further studies to identify the exact composition and structure of PD-linked α-synuclein aggregates from different areas of CNS and peripheral nervous system (PNS) should shed light on this matter.

Our results indicate that peripheral α-synuclein aggregates, in particular those derived from the SG, lack the capacity to promote α-synuclein pathology in the brain, propagate between neuronal networks or induce neurodegeneration. This observation argues against one of the currently prevalent pathogenic hypothesis of cell-to-cell transmission of α-synuclein from the periphery to the CNS [2]. However, the interpretation of our results needs some caution. In our study, we have chosen the SG as peripheral tissue because the SG is the peripheral structure that exhibits the highest burden of α-synuclein pathology and it does so invariably in all PD patients, but not control subjects [8]. However, it is possible that α-synuclein aggregates from other peripheral tissues might behave differently from a pathogenic point of view and, therefore, our results might not be generalized to all peripheral structures. This question could be addressed in subsequent studies by injecting α-synuclein aggregates derived from other peripheral regions. For instance, due to its accessibility, the gastrointestinal tract has been proposed as one of the potential earliest sites of α-synuclein pathology from where α-synuclein aggregates, initiated by exposure to a putative pathogen or infectious agent, could spread retrogradely to the brain through vagal nerve connections [3, 8–12, 23]. It appears, however, that α-synuclein can also be transported anterogradely from the brain to the stomach [27], raising the question as to whether peripheral α-synuclein aggregates really precede brain pathology in PD or may instead occur concomitantly or even follow CNS involvement [24].

While we found here that peripheral α-synuclein aggregates from SG are not pathogenic when injected into the brain, the presence of α-synuclein accumlation in peripheral tissue is often associated with peripheral autonomic denervation [8, 29], thereby suggesting a potential pathogenic effect of these aggregates in the periphery. Whether these pathological alterations in peripheral tissue are actually caused by the presence of α-synuclein remains to be determined. Because here we used postmortem SG samples derived from late PD stages, it is also possible that peripheral α-synuclein aggregates from earlier disease stages might exhibit different pathogenic capacity. This question could be eventually tested using peripheral α-synuclein aggregates from early manifest or even pre-motor PD subjects, such as those with idiopathic REM sleep behavior disorder [23, 28] or incidental Lewy body disease subjects [1, 6]. However, our previous results using SNpc-derived LB extracts demonstrated that samples derived from late stages of the disease can actually be pathogenic [20].

## Conclusions

Overall, here we demonstrate that, in contrast to CNS-derived LB [20], peripheral LB-type α-synuclein aggregates from PD patients are not pathogenic when injected to mice. The differential pathogenic capacity of CNS- and PNS-derived α-synuclein aggregates appears to be independent of the absolute amount and basic biochemical properties of α-synuclein within these fractions and may rely instead on differences in α-synuclein conformation and/or yet unrecognized brain region-specific intrinsic factors. The identification of such factors should shed light on the physiopathological role of α-synuclein in PD and open new avenues for therapeutic intervention.

## Additional file

Additional file 1: Figure S1. Representative photomicrographs of α-synuclein pathology in the SG of a 68-year old PD patient (*left*), compared to a 89-year old non-PD control subject (right), as detected by phosphorylated α-synuclein (pSyn) immunohistochemistry. Scale bar, 50 μm. (TIFF 6337 kb)
