# Supplementary figures and images for: Lack of pathogenic potential of peripheral α-synuclein aggregates from Parkinson’s disease patients
Source: Acta Neuropathol Commun. 2018 Feb 8;6:8. doi: 10.1186/s40478-018-0509-1 (PMC5806361; doi:10.1186/s40478-018-0509-1)

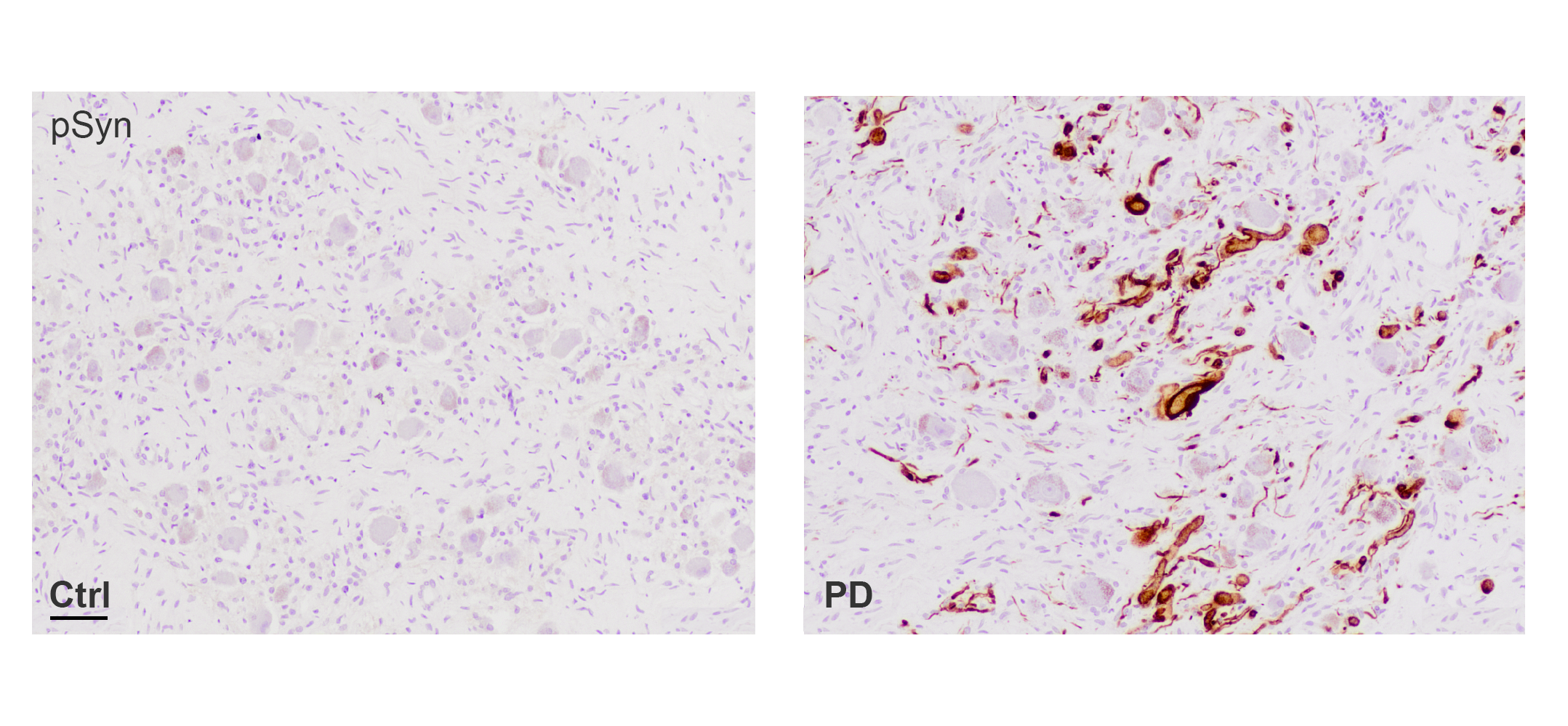

Supplement: Supplementary file 1 — Representative photomicrographs of α-synuclein pathology in the SG of a 68-year old PD patient (left), compared to a 89-year old non-PD control subject (right), as detected by phosphorylated α-synuclein (pSyn) immunohistochemistry. Scale bar, 50 μm. (TIFF 6337 kb) [file 40478_2018_509_MOESM1_ESM.tif]
